# Thrombus Tango: Hypercoagulable State With a Right Atrial Mass and Superimposed Persistent Bacteremia

**DOI:** 10.7759/cureus.61046

**Published:** 2024-05-25

**Authors:** Oshin Rai, Anvit D Reddy, Natalie Shaykh, Niyati Patel, Vanshika Tripathi, Ghania Masri

**Affiliations:** 1 Internal Medicine, University of Florida College of Medicine – Jacksonville, Jacksonville, USA; 2 Internal Medicine, University of Florida College of Medicine- Jacksonville, Jacksonville, USA

**Keywords:** pulmonary emboli, angio vac, gram positive bacteremia, right atrium thrombus, bacillus cereus

## Abstract

*Bacillus cereus* is an uncommon nosocomial bacteria, typically dismissed as a contaminant. This case is a unique scenario in which *B. cereus* bacteremia persisted despite appropriate treatment. Further investigation revealed the presence of a right atrial thrombus believed to harbor a biofilm responsible for the sustained bacteremia. Clearance of the thrombus using the AngioVac system (AngioDynamics, Inc., Latham, NY) led to the resolution of blood cultures, and subsequently, the patient was discharged with a six-week course of intravenous (IV) antibiotics.

## Introduction

*B. cereus*, characterized as a gram-positive, rod-shaped, motile bacterium, is typically encountered in environmental settings like soil, dust, and water [1]. It can be challenging to distinguish it from a contaminant in the health care setting. *B. cereus *is recognized as a nosocomial pathogen since healthcare environmental reservoirs can include air filtration, ventilation devices, lines, gloves, hands of staff, intravenous (IV) catheters, specimen collection tubes, alcohol-based hand wash solutions, and more [1]. It has commonly been linked to food poisoning, skin infections, and bacteremia [1-4]. In a population of immunosuppressed individuals, IV drug users, and neonates, it has also been documented to cause pneumonia, sepsis, and central nervous system infections [1]. Nosocomically acquired bacteremia is frequently linked to intravascular devices such as indwelling catheters, dressings, and linens [3]. A study by Sarahara et al. described a bacillus bacteremia outbreak due to infected hospital linens and concluded that bacillus should not be considered a false positive in the appropriate clinical setting [4]. The difficulty with B. cereus bacteremia is that it is typically considered a contaminant, but clinical correlation is required [1], as it has been reported to affect immunocompetent individuals and cause multi-organ dysfunction [5].

## Case presentation

We present a case of a 30-year-old female with a past medical history of end-stage renal disease (ESRD) on hemodialysis, insulin-dependent type 1 diabetes (T1DM), hypertension, and atrial flutter who presented to the hospital with several days of nausea, vomiting, abdominal pain, and body aches. Her presenting vital signs were within normal limits. Initial labs were significant for leukocytosis of 20.56 thou/cumm, carbon dioxide of 11 mmol/L, anion gap of 31 mmol/L, and glucose of 1,058 mg/dl (Table 1). She was admitted to the intensive care unit (ICU) for diabetic ketoacidosis (DKA) management and transferred to internal medicine the following day after appropriate treatment.

**Table 1:** Initial laboratory findings on presentation concern for DKA and requiring ICU admission DKA: Diabetic ketoacidosis

Complete Metabolic Panel	Value	Reference Range
Sodium	115	135 - 145 mmol/L
Potassium	5.0	3.3 - 4.6 mmol/L
Chloride	73	101 - 110 mmol/L
Carbon Dioxide	11	21 - 29 mmol/L
Urea Nitrogen	78	6 - 22 mg/dL
Creatinine	7.26	0.51 - 0.96 mg/dL
Blood Urea Nitrogen (BUN)/Creatinine Ratio	10.7	6 - 22
Glucose	1058	71 - 99 mg/dL
Calcium	8.1	8.6 - 10.0 md/dL
Total Protein	6.2	6.5 - 8.3 g/dL
Albumin	3.2	3.8 - 4.9 g/dL
Total Bilirubin	0.3	0.2 - 1.0 mg/dL
Alkaline Phosphatase	166	35 - 104 IU/L
Aspartate Transaminase (AST)	15	14 - 33 IU/L
Alanine Transaminase (ALT)	11	10 - 42 IU/L
Anion Gap	31	4 - 16 mmol/L
Estimated Glomerular Filtration Rate (eGFR) (EGFR)	7	≥ 60 mL/min/1.73M2
Hemoglobin A1C	10.2	4.8 - 5.9 %
Complete Blood Count and Differential		
White Blood Cell (WBC)	20.5	4.0 - 10.0 x10^3^/µL
Red Blood Cell (RBC)	4.23	4.0 - 5/2 x10^6^/µL
Hemoglobin	11.4	12.0 - 16.0 g/dL
Hematocrit	35.5	35.0 - 45.0 %
Mean Corpuscular Volume (MCV)	83.9	78.0 - 100.0 fl
Mean Corpuscular Hemoglobin (MCH)	27.0	26.0 - 34.0 pg
Mean corpuscular hemoglobin concentration (MCHC)	32.1	31.0 - 36.0 g/dL
Red Cell Distribution Wiidth (RDW)	14.9	11.0 - 14.6%
Platelet Count	294	150 - 450x10^3^/µL
Mean Platelet Volume (MPV)	11.8	9.5 - 12.2 fl
Neutrophil %	93	34 - 73%
Bands %	28.4	0 - 10%
Lymphs %	3	25 - 45%
Monocytes %	2	2 - 6%
Metamyelocytes %	5	≤ 0%
Myelocytes %	1	≤ 0%
Promyelocytes %	0	≤ 0%
Neutrophil Absolute	19.29	1.4 - 7.5x10^3^/µL
Lymphocyte Absolute	0.53	0.7 - 3.1x10^3^/µL
Monocytes Absolute	1.30	0.1 - 0.9x10^3^/µL
Immature Granulocytes Absolute	0.06	≤ 0.0x10^3^/µL

**Figure 1:**
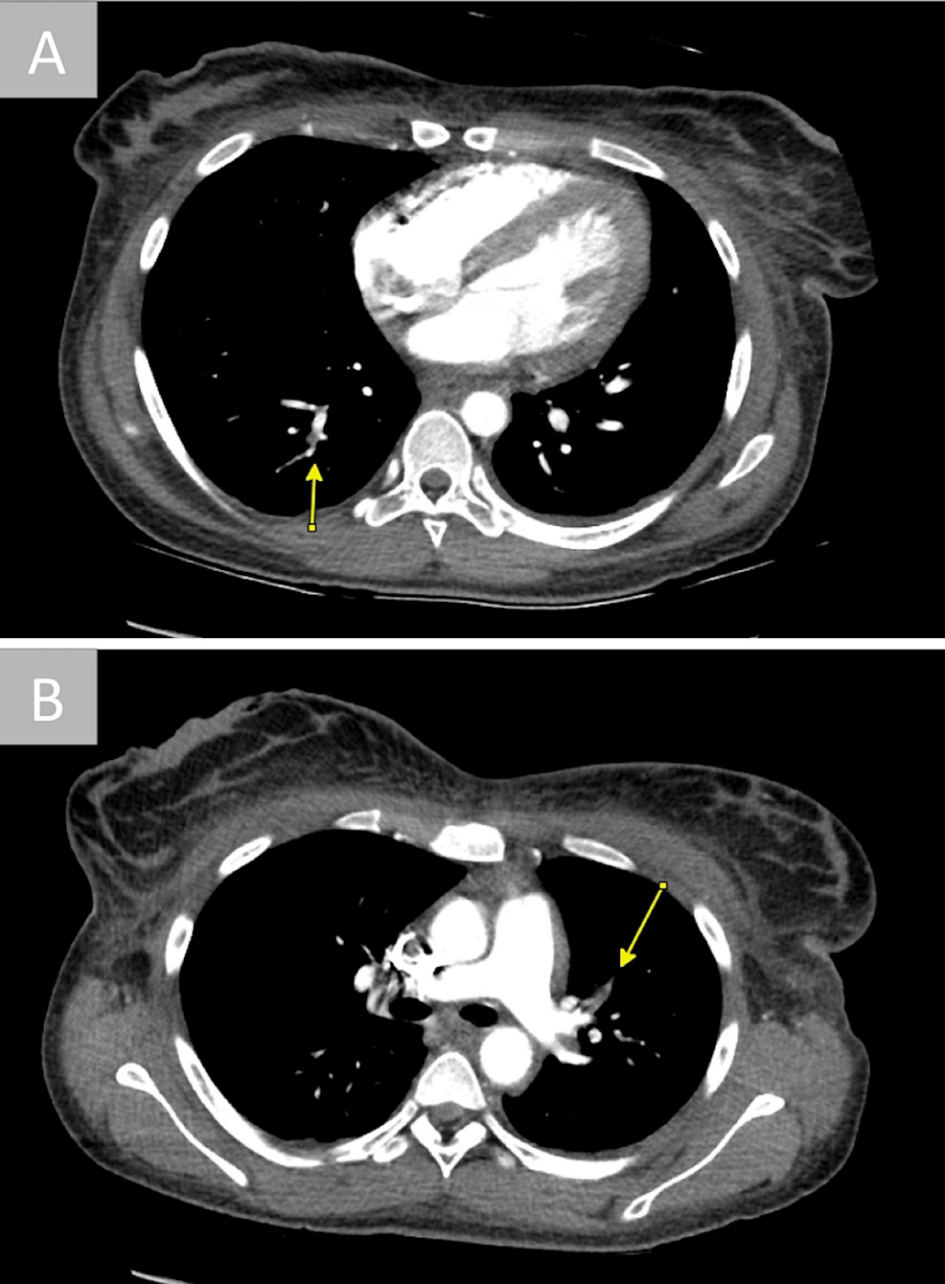
Computed tomography of the chest with pulmonary embolism protocol (CTA PE) The images show (A) right lower lobe posterior basilar segmental and subsegmental and (B) left upper lobe anterior segmental and subsegmental pulmonary emboli.

During her ICU course, an infectious workup was obtained to determine the etiology of her DKA. Blood cultures returned positive on hospital day two for *B. cereus* in two sets of blood cultures. Her tunneled catheter was removed, with the catheter tip tip testing negative. To investigate her abdominal pain and source of bacteremia further, a computed tomography (CT) scan of the abdomen and pelvis was obtained and revealed diffuse mural thickening of the bilateral collecting system, ureters, and urinary bladder, suggesting the bacteremia originated from a urinary source. The scan also noted questionable filling defects within the sub-segmental branches of the right pulmonary artery. A subsequent CT angiography pulmonary embolism protocol revealed both right lower lobe and left lower lobe segmental and subsegmental pulmonary emboli (Figure 1) without evidence of right heart strain. She was continued on IV vancomycin, per infectious disease recommendations for concern of possible seeding of right atrial mass, and a heparin drip.

**Figure 2:**
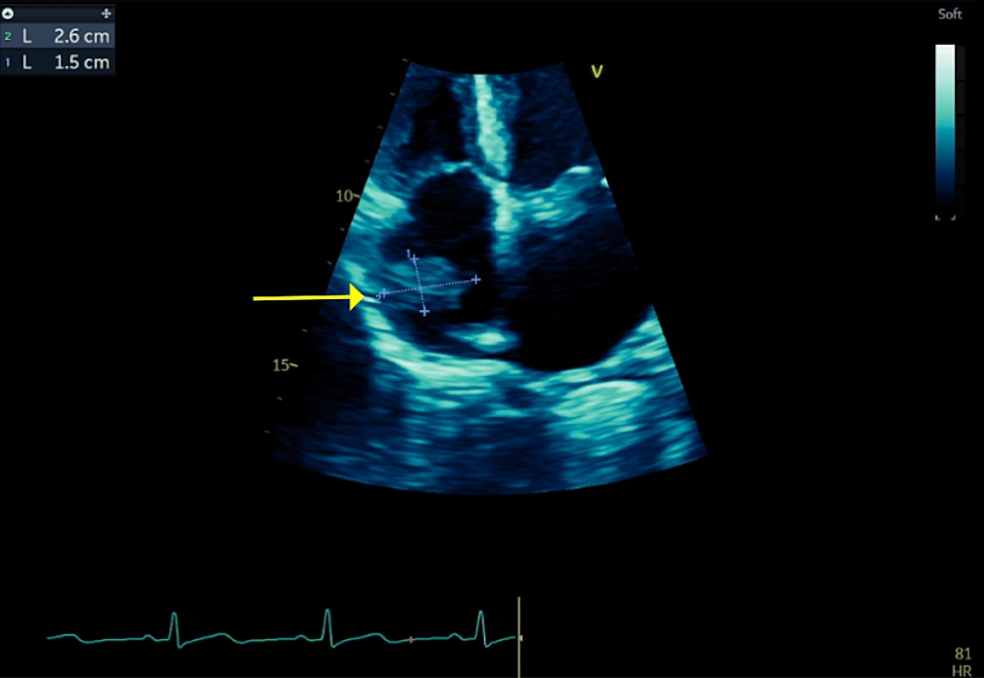
Transthoracic echocardiogram (TTE) image The yellow arrow shows a 2.6 cm by 1.5 cm right atrial mass without clear valvular vegetations.

**Figure 3:**
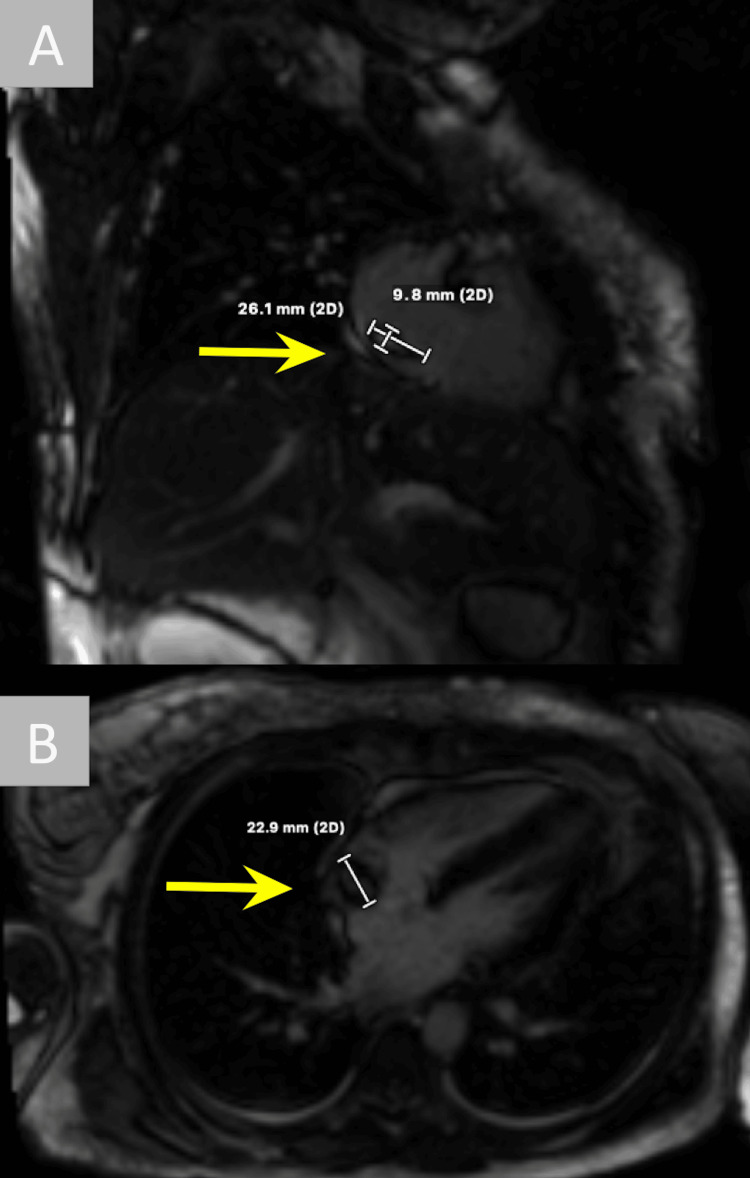
Cardiac magnetic resonance imaging (cMRI) images Cardiac magnetic resonance imaging (cMRI) revealed a right atrial peripherally hypointense lesion measuring 2.6 cm x 2.3 cm x 0.9 cm, with a gray etched core close to the crista terminalis and homogeneous low signal.

To rule out endocarditis, transthoracic echo (TTE) was performed and showed an estimated 2.6 cm by 1.5 cm right atrial mass without clear valvular vegetations (Figure 2). It was unclear if this mass was infective, given the bacteremia, or thrombotic, given the known pulmonary embolisms. Additionally, lower on the differential was atrial myxoma or another cardiac tumor. Cardiology recommended cardiac magnetic resonance imaging (cMRI) to further characterize the mass, which more clearly revealed a right atrial peripherally hypointense lesion measuring 2.6 cm x 2.3 cm x 0.9 cm, with a gray etched core close to the crista terminalis and homogeneous low signal, compatible with thrombus (Figure 3).

**Figure 4:**
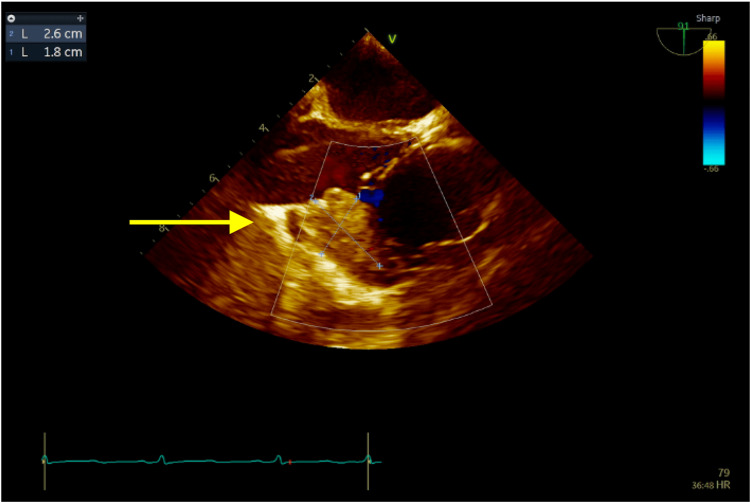
Transesophageal echocardiogram (TEE) image The image shows an irregularly shaped right atrial 2.6 cm x 2.3 cm x 1.0 cm (anterior-posterior, transverse, craniocaudal) peripherally hypointense lesion with a gray etched core close to the crista terminalis. On phase-sensitive inversion recovery sequence, the entire lesion demonstrates homogeneous low signal compatible with thrombus. There is no lesional contrast enhancement.

Despite therapy with appropriate IV antibiotics, three additional sets of blood cultures remained positive. Per the recommendations of infectious disease, an epidural abscess workup with imaging of the thoracic and pelvis was completed, which came back negative. A thorough physical exam was also grossly unremarkable. Cardiology performed a transesophageal echocardiogram (TEE) guided trans-catheter vacuum-assisted aspiration of intracardiac mass using AngioVac ((AngioDynamics, Inc., Latham, NY). There were noted to be three echo-densities in the right atrium, including a fixed 2.6 cm x 2.3 cm x 1.0 cm mass at the inferior and posterior aspect of the right atrium free wall (Figure 4), a smaller attached mass of 1.7 cm by 0.4 cm, and a mobile 1.7 cm x 0.6 cm mass. The AngioVac evacuated the mobile mass and the attached masses remained in place. Repeat TTE showed fixed echogenic masses in place without any mobile masses observed.

**Figure 5:**
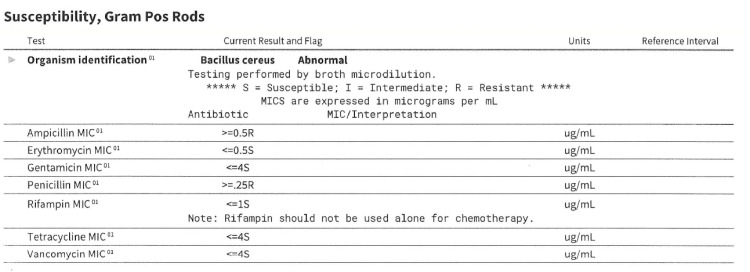
Susceptibility testing report done through broth microdilution

Her blood cultures were cleared after nine days of initial positive results. In our study, we utilized Matrix-Assisted Laser Desorption/Ionization-Time of Flight (MALDI-TOF) mass spectrometry to identify the bacterial isolate of *B. cereus*. Further susceptibility testing was performed by broth microdilution, with results in Figure 5. 

She was discharged home with a peripherally inserted central catheter (PICC) line with plans for a six-week course of vancomycin, anticoagulation with apixaban, and close outpatient follow-up. She did well with no reoccurrence of the bacteremia.

## Discussion

*B. cereus* has been implicated in infections with a history of IV drug use, central venous catheters, intracardiac hardware or mucosal injuries in neutropenic patients [6]. This case was unusual and remained ambiguous until further assessment was undertaken, as the patient lacked the typical risk factors associated with gram-positive bacteremia or endocarditis with no history of IV drug use or prosthetic heart valves. Additionally, her most likely presumed source of infection, the catheter tip, tested negative. However, her poorly managed T1DM might have increased her susceptibility to systemic infections [7]. In the initial evaluation, the TTE findings were inconclusive regarding the presence of a right atrial thrombus or infective endocarditis. In our case, these findings were contradictory, but given the initial TTE findings and incidental pulmonary embolisms, it was decided to place the patient on anticoagulation with a heparin drip. Currently, there are no specific guidelines for the sole treatment of endocarditis and initiating anticoagulation, as it can be challenging with the increased risk of embolic or hemorrhagic complications [8]. 

Differentiating between a thrombus and vegetation can be difficult, but they have some distinct differences. A thrombus usually will have a smooth surface, and the sign of an older thrombus is its adherence to the vascular endothelium, making it difficult to detach [9]. In comparison, vegetations caused by infective endocarditis have an uneven appearance on echo, and their fragile structure predisposes them to an increased likelihood of rupture or embolism. Our patient had atrial fibrillation as a risk factor to support thrombus. On the other hand, she also had gram-positive bacteremia, suggesting endocarditis. Endocarditis is an uncommon diagnosis with an annual incidence of three to seven per 100,000 people per year, yet it poses a high mortality [10]. Right-sided infective endocarditis often occurs in the setting of intravenous drug use and intravascular devices such as pacemakers and tunneled dialysis catheters [11]. In the case of our patient, the vegetations were not valvular and the cardiac MRI further expanded on its appearance. Typical microbes that lead to endocarditis are aerobic gram-positive and gram-negative bacteria, with anaerobic bacteria being rare [3].

Although uncommon, we postulate that her bacteremia came from a urinary source and proliferated, leading to bacteremia, supported by the catheter tip testing negative. The persistence of the bacteremia can be attributed to *B. cereus*' ability to form biofilms, leading to microcolony formation and the aggregated effect of planktonic bacteremia, creating more biofilms [1]. Antibiotic therapy can target planktonic bacteria but sessile bacteria are protected and are the reason spore survival and replication are possible in a hospital setting [1]. IV antibiotics remain the mainstay of treatment with *B. cereus *sensitive to aminoglycosides, clindamycin, erythromycin and vancomycin [3,10]. In cases such as ours, where there was resistant bacteremia (positive cultures greater than five days) and a large thrombus (mobile mass greater than 10 mm), a surgical evacuation was indicated as a class IIa recommendation [10]. AngioVac, an alternative to invasive surgery, has been utilized as a novel treatment for right-sided endocarditis, with case reports documenting its success in both evacuating vegetations or thrombi and contributing to bacteremia clearance [11]. Similarly, in our patient, the AngioVac removed the mobile mass, which led to the clearance of the bacteremia.

## Conclusions

This elusive case of *B. cereus* bacteremia with superimposed right atrial thrombus created difficult management decisions. We hypothesize an atypical urinary source of the bacteremia and that the right atrial mass became a nidus of persistent bacteremia. She clinically improved after removal of the thrombus through AngioVac. Further research needs to be done regarding *B. cereus *infections and its complications.
